# CAR T therapies in multiple myeloma: unleashing the future

**DOI:** 10.1038/s41417-024-00750-2

**Published:** 2024-03-04

**Authors:** Mohsen Sheykhhasan, Amirhossein Ahmadieh-Yazdi, Rosario Vicidomini, Naresh Poondla, Hamid Tanzadehpanah, Ashkan Dirbaziyan, Hanie Mahaki, Hamed Manoochehri, Naser Kalhor, Paola Dama

**Affiliations:** 1 Cellular and Molecular Research Center, Qom University of Medical Sciences, Qom, Iran; 2 Stem Cell Biology Research Center, Yazd Reproductive Sciences Institute, Shahid Sadoughi, University of Medical Sciences, Yazd, Iran; 3 Section on Cellular Communication, Eunice Kennedy Shriver National Institute of Child Health and Human Development (NICHD), National Institutes of Health (NIH), Bethesda, MD USA; 4 Icahn School of Medicine at Mount Sinai, New York, USA; 5 Antimicrobial Resistance Research Center, Mashhad University of Medical Sciences, Mashhad, Iran; 6 Department of Microbiology, Faculty of Medicine, AJA University of Medical Sciences, Tehran, Iran; 7 Vascular & Endovascular Surgery Research Center, Mashhad University of Medical Sciences, Mashhad, Iran; 8 The Persian Gulf Marine Biotechnology Research Center, The Persian Gulf Biomedical Sciences Research Institute, Bushehr University of Medical Sciences, Bushehr, Iran; 9 Department of Mesenchymal Stem Cells, Academic Center for Education, Culture and Research, Qom, Iran; 10 School of Life Sciences, University of Sussex, Brighton, BN1 9QG UK

**Keywords:** Myeloma, Cancer

## Abstract

In recent years, the field of cancer treatment has witnessed remarkable breakthroughs that have revolutionized the landscape of care for cancer patients. While traditional pillars such as surgery, chemotherapy, and radiation therapy have long been available, a cutting-edge therapeutic approach called CAR T-cell therapy has emerged as a game-changer in treating multiple myeloma (MM). This novel treatment method complements options like autologous stem cell transplants and immunomodulatory medications, such as proteasome inhibitors, by utilizing protein complexes or anti-CD38 antibodies with potent complement-dependent cytotoxic effects. Despite the challenges and obstacles associated with these treatments, the recent approval of the second FDA multiple myeloma CAR T-cell therapy has sparked immense promise in the field. Thus far, the results indicate its potential as a highly effective therapeutic solution. Moreover, ongoing preclinical and clinical trials are exploring the capabilities of CAR T-cells in targeting specific antigens on myeloma cells, offering hope for patients with relapsed/refractory MM (RRMM). These advancements have shown the potential for CAR T cell-based medicines or combination therapies to elicit greater treatment responses and minimize side effects. In this context, it is crucial to delve into the history and functions of CAR T-cells while acknowledging their limitations. We can strategize and develop innovative approaches to overcome these barriers by understanding their challenges. This article aims to provide insights into the application of CAR T-cells in treating MM, shedding light on their potential, limitations, and strategies employed to enhance their efficacy.

## Introduction

Multiple myeloma (MM) is the second most common hematological cancer, characterized by the abnormal proliferation of plasma cells and contributes to 2% of cancer-related deaths in the United States [1]. Initially addressed with melphalan, advances in disease understanding have transformed the therapeutic landscape. The introduction of immunomodulatory drugs (thalidomide, lenalidomide, and pomalidomide), proteasome inhibitors (bortezomib, carfilzomib, and ixazomib), histone deacetylase inhibitors, and FDA-approved monoclonal antibodies (daratumumab and elotuzumab), has broadened treatment options for MM patients [2–4]. Despite these advancements, multiple myeloma remains predominantly incurable, especially for high-risk patients who do not benefit from the current treatment options [4]. In this context, immunotherapy-based medications present promising advancements in the treatment of multiple myeloma, encompassing checkpoint inhibitors, antibody-drug conjugates, bispecific T cell engagers (BiTEs), and adoptive T cell therapy (ACT) [5–9]. A particularly promising immunotherapeutic avenue is Chimeric Antigen Receptor (CAR) T-cell therapy, which has shown remarkable results in B-cell malignancies [10, 11] FDA-approved CAR T-cell therapies, such as tisagenelcleucel/Kymriah (Novartis) and Brexucabtagene Autoleucel/Tecartus (Kite Pharma) for acute lymphoblastic leukemia (ALL), axicabtagene ciloleucel/Yescarta (Gilead/Kite) and lisocabtagene maraleucel/Breyanzi (Bristol Myers Squibb), and Idecabtagene Vicleucel/Abecma (Bristol Myers Squibb and bluebird bio) for MM, underscore the efficacy of CAR T-cell therapy in cancer treatment [12, 13]. A notable milestone was achieved in February 2022 with FDA approval granted to ciltacabtagene autoleucel/Carvykti (Janssen Biotech) for MM treatment [13]. Immunotherapy, particularly CAR T-cell therapy, emerges as a promising frontier in the ongoing pursuit of effective multiple myeloma treatment.

## CAR T-cell therapy

**Fig. 1:**
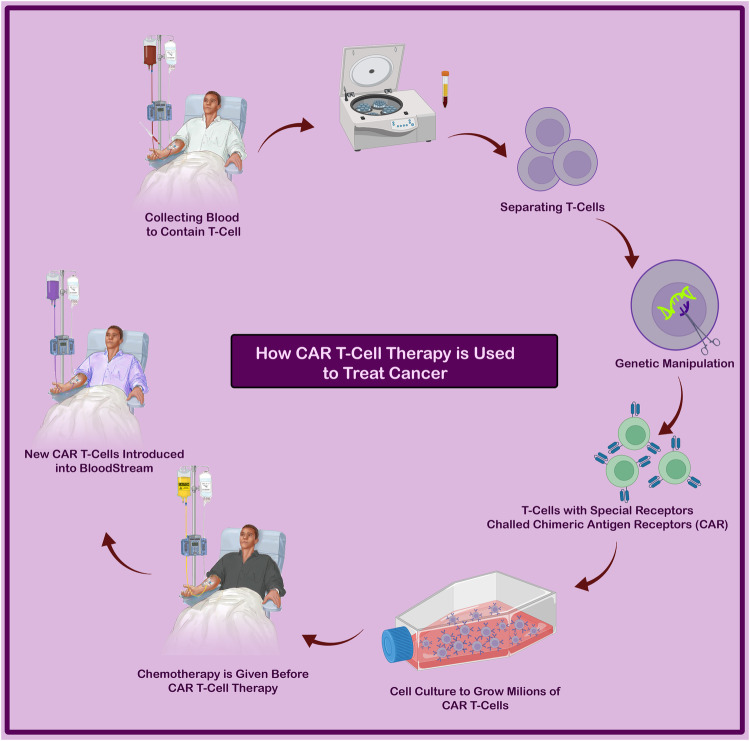
Overview of autologous CAR T cell manufacturing. The production of autologous CAR T cells begins with a patient’s leukapheresis, followed by T cell enrichment and activation. To promote the introduction and perhaps permanent integration of the CAR transgene, activated T cells are transduced (e.g., with a lentiviral vector). T cells that have been genetically engineered are then grown in either static or dynamic culture, cryopreserved, and reintroduced into the patient.

**Fig. 2:**
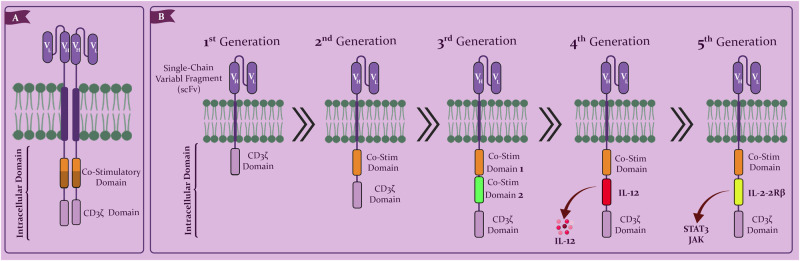
Structure of five generations of CAR T-cells. **A** The structure of CAR T-cells. **B** The first-generation CAR signaling domain has solely a CD3-derived signaling domain. A co-stimulatory domain is also present in second-generation CARs. CARs of the third generation have two co-stimulatory domains. Fourth-generation CAR T cells express chemokines such as IL-12 when activated. Fifth-generation CARs have a unique co-stimulatory domain that activates specific signaling pathways, such as JAK/STAT3.

Chimeric Antigen Receptors (CARs) are fusion proteins designed to target specific antigens expressed on cell surfaces. The first chimeric receptor was developed by Eshhar’s group at the Weizmann Institute of Science in 1989 [14]. Since their initial development, CAR T-cells have rapidly evolved through various generations. CARs consist of three essential domains: extracellular, transmembrane, and intracellular domains [15]. The intracellular domain plays a crucial role in signaling T lymphocytes, enabling the killing of malignant cells independent of the human leukocyte antigen (HLA) [16]. CAR T therapy has revolutionized cancer treatment by offering personalized treatment based on the specific type of cancer and the patient’s requirements. This approach involves genetically modifying the patient’s own T cells, which are then able to recognize and bind to tumor antigens. After the modified T cells undergo proliferation, they are infused back into the patient’s body to target and eliminate cancer cells (Fig. 1) [17]. The extracellular domain, transmembrane domain, and intracellular domain are the three major components of a CAR [18]. The extracellular domain consists of a single-chain variable fragment (scFv), which is a fusion protein of the antibody’s light and heavy chains’ variable regions. This domain is connected to the transmembrane domain via a spacer and further linked to the intracellular signaling domain, leading to cancer cell cytolysis [16]. The extracellular scFv facilitates the attachment of CAR T-cells to specific cells, while the intracellular domain aids in T cell activation [19]. Within the intracellular signaling domain, there are primary stimulatory and secondary costimulatory domains (Fig. 2).

CARs targeting MM antigens often utilize the CD3zeta intracellular stimulatory domain, which contains immunoreceptor tyrosine activation motifs (ITAMs) that generate “signal 1”. CAR T-cells are categorized into various subgroups based on the presence of costimulatory domains (Fig. 2B) [20]. First-generation CARs, lacking costimulatory domains, were not efficient enough for clinical use. However, NKG2D CARs are an exception due to their natural ligands, possessing an endogenous costimulatory domain that functions independently [21]. Second-generation CARs, incorporating either CD28 or 4-1BB (CD137) costimulatory domains, are the most commonly employed in multiple myeloma. While 4-1BB CARs exhibit a memory stem cell-like phenotype and longer persistence, CD28-based cells are more potent with greater growth capacity [22], suggesting a predominance of 4-1BB in CAR designs. Other costimulatory domains, such as OX40 (CD134), CD27, inducible T cell costimulatory (ICOS), CD40, and MYD88, have been developed, but they have only been investigated at the preclinical level [20]. Third-generation CARs with two or more costimulatory domains show increased effectiveness and persistence. However, designing and developing such CARs is more complex, and they are still in the early stages of development, making them less effective than single-antigen targeting. Moreover, if tumor cells lose one of the antigens, the costimulatory CAR is not triggered [12, 20]. In addition, fourth- and fifth-generation MM antigen-targeted CARs have been developed, capable of producing immunomodulatory molecules (IL-7, CCL19) in response to antigen stimulation [23]. These CARs interact with tumor cells, forming an immune synapse that leads to target cell killing through various pathways, including the release of cytotoxic molecules (perforins, granzymes), induction of apoptosis via the Fas-Fas ligand molecular pathway, and cytokine production. These pathways also stimulate lymphocyte proliferation and activate other immune cells [24].

## CAR T-cell therapy in MM

**Fig. 3:**
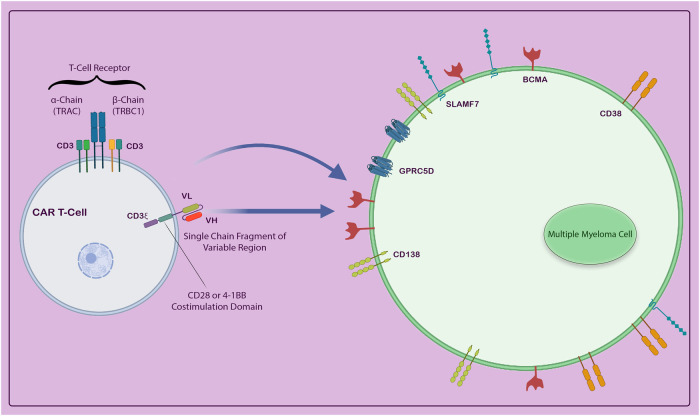
Potential target antigens. Antigens associated with multiple myeloma, including CD19, CD38, CD138, BCMA (B-cell maturation antigen), Kappa (κ) light chain, SLAM7, NKG2D, and GPRC5D, can be exploited to create particular CAR-T cells efficiently.

Surprisingly, CAR T-cell therapy has emerged as a widely utilized treatment for various hematological cancers, including acute and chronic leukemia, lymphoma, and multiple myeloma [25, 26]. Despite initial concerns regarding its efficacy and safety, CAR T-cell therapy has proven to be one of the most promising and potent treatment options available. One prevalent form of CAR T-cells targets CD19 and has been extensively developed for the treatment of leukemias and lymphomas [22]. In addition, CAR T-cells targeting CD22 and CD20 have shown promise in treating ALL [27] and relapsed/refractory NHL, respectively [28]. CAR T-cells can also target other antigens in various cancers, such as CS-1, CD30, CD38, and CD138Prior studies have successfully identified a range of target antigens present on cancer cells. These antigens are pivotal for the development of specialized CAR T-cells. In the context of Multiple Myeloma, notable antigens such as CD19, CD38, CD138, BCMA (B-cell Maturation Antigen), Kappa (κ) light chain, SLAMF7, NKG2D, and GPRC5D have been identified as effective targets for CAR T-cell therapy (Fig. 3). BCMA, a protein present in high concentrations on a small subset of healthy blood cells and multiple myeloma cells, is the most extensively studied CAR target for myeloma. BCMA, a tumor necrosis factor receptor superfamily member 17 (TNFRSF17), is preferentially expressed on plasma cells but not on CD34+ hematopoietic stem cells, making it a promising antigenic target [29]. The binding and activation of ligands such as B-cell activating factor (BAFF) and a proliferation-inducing ligand (APRIL) to BCMA enhance plasma cell development and proliferation in the bone marrow [30]. Although BCMA expression is heterogeneous [31], it is found in all multiple myeloma cells, and its overexpression has significant prognostic implications [32]. The first BCMA-directed CAR was developed a decade ago and demonstrated effective targetability in preclinical studies [29]. Subsequently, the first-in-human Phase I clinical trial to evaluate the efficacy of BCMA-targeted CAR T-cells in relapsed/refractory multiple myeloma (RRMM) was conducted (NCT02215967) [33, 34]. The trial reported an overall response rate (ORR) of 81%, with 63% achieving very good partial (VGPR) or complete response (CR) [33, 34]. Consequently, there has been a widespread effort to develop new anti-BCMA CARs and optimize existing ones. This article explores the development of CAR T-cells against these antigens and discusses the efficacy of CAR T-cell therapy in multiple myeloma.

## bb2121 anti-BCMA CAR T cell

Following the promising results of preliminary studies, a Phase I clinical trial was conducted, involving 33 patients with Relapsed and Refractory Multiple Myeloma (RRMM) who were administered anti-BCMA CAR T-cells (known as bb2121) featuring anti-BCMA ScFv, CD3, and 4-1BB domains (NCT02658929) [35]. This trial comprised two distinct phases. In the initial dose-escalation phase, patients received bb2121 (manufactured by Celgene) at varying doses: 50 × 10^6^, 150 × 10^6^, 450 × 10^6^, or 800 × 10^6^ CAR + T cells. Subsequently, in the dose expansion phase, patients received a total of 150 × 10^6^ to 450 × 10^6^ CAR + T cells [35]. Encouragingly, the median duration without disease progression was 11.8 months, with a 95% confidence interval ranging from 6.2 to 17.8 months. Out of the 16 patients who attained a partial response or superior outcome, all underwent rigorous assessment for minimal residual disease (MRD), and none displayed any evidence of MRD presence, with sensitivity down to 104 nucleated cells. Remarkably, among the patients assessed, an impressive overall response rate (ORR) of 85% was documented. However, it is crucial to note that this therapeutic approach was not without its challenges. Notably, a substantial proportion of patients, specifically 76%, experienced cytokine release syndrome (CRS), a severe systemic inflammatory response frequently observed in CAR T-cell therapies. Symptoms of CRS varied in intensity and included fever, fatigue, nausea, muscle aches, difficulty breathing, low blood pressure, and in severe cases, organ dysfunction. In addition, 42% of patients encountered neurotoxicity as an adverse event [35].

In the Phase 1 CRB-401 trial (NCT02658929), an updated analysis of ide-cel’s application in Relapsed and Refractory Multiple Myeloma (RRMM) revealed a median progression-free survival (PFS) of 8.8 months, alongside an overall survival (OS) of 34.2 months. Notably, a discernible dose-dependent effect was observed, with more favorable responses and survival outcomes seen when administering ≥150 × 10^6^ CAR + T cells [36].

A pivotal phase II single-arm KarMMa clinical trial (NCT03361748) was conducted to confirm the efficacy and safety of idecabtagene vicleucel versus conventional care (CC). Patients with RRMM who were resistant after at least three previous regimens including a proteasome inhibitor, an immunomodulatory agent, and an anti-CD38 antibody were enrolled [37, 38]. The median progression-free survival (PFS) was reported as 8.6 months, while the overall survival (OS) was 24.8 months [39, 40].

Another update on the use of ide-cel in RRMM indicated that patients with triple-class exposed (TCE) RRMM who received a single infusion of ide-cel experienced significant improvements across various health-related quality of life ((HRQoL) categories during a 24-month follow-up [41]. The phase II KarMMa trial, which utilized ide-cel, demonstrated efficacy in high-risk, heavily pretreated RRMM patients. The trial showed an ORR of 73%, CR rate of 33%, and time to first response of one month. In 79% of individuals with CR, minimal residual disease negativity was achieved, with a median overall survival (OS) of 24 months and a median progression-free survival (PFS) of 8.6 months. The probability of progression-free survival increased with greater response depth and was dose-dependent. The most common adverse effects included grade 3 cytopenias, any-grade CRS occurring in 84% of cases with a time to onset of 1 day, and any-grade neurotoxicity in 18% of cases with a time to onset of 2 days, which is consistent with expectations for CAR T-cell therapy [41].

The Food and Drug Administration (FDA) approved idecabtagene vicleucel (Abecma), ide-cel, in March 2021.The approval was based on a single-arm study involving 100 adult patients with RRMM who were treated with ide-cel. The study evaluated patients’ overall response rate (ORR), complete response rate (CRR), and response durability (DOR) following treatment with ide-cel after lymphodepleting treatment with cyclophosphamide and fludarabine [42].

Among the 100 patients in the efficacy evaluable sample, the ORR was 72% (95% confidence interval [CI]: 62–81), with a strict CR rate of 28% (95% CI: 19–38). After a median follow-up of 10.7 months, the median DOR in responders (partial response or better) was 11 months (95% CI: 10.3–11.4), and in patients who achieved strict CR, it was 19 months (95% CI: 11.4 months, not estimable [NE]). Safety evaluation of 127 patients revealed that serious adverse events occurred in 67% of cases. Neurologic toxicities and grade 3 or higher cytokine release syndrome occurred in 9% and 4% of cases, respectively, leading to the implementation of a Risk Assessment and Mitigation Plan. Macrophage activation syndrome/hemophagocytic lymphohistiocytosis occurred in 4% of cases, resulting in two deaths. Long-term cytopenia requiring hematopoietic rescue was observed in 2% of patients (3/127), including two deaths [42].

The long-term results from the CRB-401 study, last updated in August 2023 by Lin et al., continue to demonstrate the safety and tolerability of ide-cel in treating heavily pretreated patients with relapsed/refractory multiple myeloma (RRMM). Ide-cel, at target dose levels of ≥150 × 10^6^ CAR + T cells, showed deep and durable responses, presenting a favorable clinical benefit-risk profile. The study also identified translational correlates for durable response to ide-cel, potentially advancing autologous CAR T cell therapy by helping select patients likely to have the most durable responses. Furthermore, the efficacy of ide-cel is being explored in several ongoing trials for earlier stages of myeloma treatment (KarMMa-2, KarMMa-3, KarMMa-4, and KarMMa-7) [43–45].

## bb21217 anti-BCMA CAR T cell

One notable advancement in CAR T-cell therapy for the treatment of MM is the development of bb21217, an anti-BCMA CAR T-cell therapy (NCT03274219), building on the investigational therapy bb2121 [45–47]. The treatment incorporates the same single-chain variable fragment (scFv), 4-1BB costimulatory motif, and CD3-zeta T cell activation domain found in bb2121. However, it also includes the phosphoinositide 3 kinase inhibitor bb007 during its ex vivo culture process. This addition is specifically aimed at enhancing the composition of the drug product, particularly enriching it with T cells that exhibit a memory-like phenotype, reducing the number of highly differentiated or senescent T cells. CRB-402 (NCT03274219) is a phase 1 clinical trial evaluating bb21217 in patients with relapsed or refractory multiple myeloma (RRMM) [47, 48]. The trial involves patients who have undergone at least three prior treatments and tests the safety, pharmacokinetics, efficacy, and duration of bb21217’s effects. This next-generation CAR T-cell therapy has shown promise in early clinical trials. The main objective of the trial was to evaluate adverse events and dose-limiting toxicities. Other objectives included evaluating the quality and duration of the clinical response, monitoring minimal residual disease, and measuring progression-free and overall survival. The trial also aimed to quantify the CAR+ cells in the blood post-treatment [47, 48]. As of February 16, 2021, in the multicenter phase I study (NCT03274219) [45], 72 patients with RRMM were enrolled and treated in doses ranging from 150 to 450 × 10^6^ CAR + T cells. The median follow-up period was 9 months. These patients had undergone an average of 6 prior treatments, 68% being triple refractory. A common side effect observed was cytokine release syndrome (CRS), affecting 75% of patients, mostly of mild to moderate severity, with one severe case and two deaths. CRS typically began 2 days after treatment, managed with tocilizumab and corticosteroids. Neurotoxicity was noted in 15% of patients, occurring around 7 days post-treatment. In terms of efficacy, CAR + T cells remained detectable in 81% of patients at 6 months and 60% at 12 months post-infusion. Among patients achieving complete response, 93% were minimal residual disease (MRD) negative. The analysis also found that patients with higher levels of CD8 + CAR + T cells displaying less differentiated, more proliferative characteristics had significantly longer response durations [49].

The results of the study revealed encouraging outcomes, particularly in terms of CAR T-cell expansion within the patients. Notably, significant growth of CAR T-cells was observed, indicating successful engraftment and proliferation of the modified cells in the treated individuals. This is an important milestone as robust expansion of CAR T-cells is crucial for the therapy to target and eliminate cancerous cells effectively. The study concludes that while adverse events align with typical CAR T cell therapy toxicities, the efficacy results, including a median duration of response of 17 months, are promising. The findings support the hypothesis that the memory-like T cell phenotype associated with bb21217 leads to prolonged response durations, and highlight the encouraging therapeutic potential of bb21217 in treating of RRMM [49].

## LCAR-B38M anti-BCMA CAR T cell

A chimeric antigen receptor T cell product known as LCAR-B38M has been developed to target two distinct B cell maturation antigen epitopes, incorporating [two VHH binding domains [50]. The LCAR-B38M lentiviral construct utilizes a tandem heavy chain-based CAR llama-derived to enhance avidity [50]. In addition, it contains a 4-1BB/CD3 intracellular signaling domain and two single-domain antibodies that specifically target BCMA [50].

In a multicenter study (NCT03090659) [50], the efficacy of a bispecific CAR T-cell therapy (LCAR-B38M) was evaluated in advanced RRMM patients, targeting the VHH1 and VHH2 epitopes of BCMA. The preliminary results of the first 57 patients treated with LCAR-B38M anti-BCMA CAR T-cells at The Second Affiliated Hospital of Xi’an Jiaotong University showed an overall response rate (ORR) of 88 percent and a CR rate of 68 percent [50]. In addition, 63% of these patients achieved negative minimal residual disease (MRD) status. However, side effects, including CRS, leukopenia, thrombocytopenia, and pyrexia, were documented [50].

In 2022 the authors reported long-term safety and efficacy data from a median follow-up of 4 years. A phase 1, single-arm, open-label study named LEGEND-2 was conducted at four authorized locations in China [51]. Over a four-year period, LCAR-B38M showcased significant effectiveness, achieving an overall response rate (ORR) of 87.8%, a minimal residual disease (MRD) negativity rate of 67.6%, and a median progression-free survival (PFS) of 18 months in relapsed/refractory multiple myeloma (RRMM). Interestingly, despite a declining trend in PFS, the overall survival (OS) curve showed signs of stabilizing, indicating effective response to later treatments in progressing patients. Furthermore, this treatment demonstrated a manageable safety profile, balancing efficacy with tolerability [51].

## P-BCMA-101 anti-BCMA CAR T cell

P-BCMA-101 represents an exciting breakthrough in the field of CAR T-cell therapy. This innovative treatment utilizes a novel design that incorporates fully-humanized anti-BCMA ScFv extracellular domains, CD3 signaling domains, and 4-1BB signaling domains. By utilizing these components, P-BCMA-101 CAR T-cells exhibit a more potent structure, enabling enhanced targeting and destruction of cancer cells expressing BCMA (B-cell maturation antigen) [52].

One of the key advantages of P-BCMA-101 is its use of a transposon system instead of viral vectors. This approach not only enhances the expression levels of CAR T-cells but also minimizes the risk of immunogenicity, thus improving the overall safety profile of the therapy. By bypassing the use of viral vectors, P-BCMA-101 CAR T-cells can be generated with high efficiency, providing a reliable and scalable manufacturing process [52].

To evaluate the efficacy and safety of P-BCMA-101 CAR T-cells, a phase I clinical trial (NCT03288493) [52] was conducted in patients with RRMM. In this trial, a total of twelve patients were enrolled, and their responses were closely monitored. Encouragingly, approximately one out of twelve patients achieved a CR, indicating a significant reduction or even eradication of cancer cells [52, 53]. It is worth noting that during the trial, one patient experienced grade 2 CRS. The incidence of CRS in this trial was relatively low, suggesting that P-BCMA-101 CAR T-cells demonstrate a favorable safety profile [52, 53].

These findings from the phase I clinical trial provide promising preliminary evidence of the potential effectiveness and safety of P-BCMA-101 CAR T-cell therapy in patients with RRMM.

As of June 30, 2021, 90 patients were treated with P-BCMA-101 at various dose levels, both as a single agent and in combination with Rituximab (Rit) or Lenalidomide (Len) [54]. The patients, mostly in their early sixties, had extensively received prior treatments, including proteasome inhibitors (PI) and immunomodulatory drugs (IMiD). The therapy showed a favorable safety profile with no significant toxicities. Mild to moderate cytokine release syndrome (CRS) was noted in 25% of patients and neurological side effects in 7%. The most common side effects included low blood counts, infections, and general symptoms. The response rate to the therapy was high (73% with Rit and 71% with Len). Due to its safety, 25% of the treatments were administered on an outpatient basis. The development of P-BCMA-101 CAR T-cells brings hope for improving the treatment outcomes and quality of life for patients with multiple myeloma, paving the way for a new era of personalized and targeted cancer therapies [54].

## MCARH171 anti-BCMA CAR T cell

In a phase I dose-escalation trial (NCT03070327) [55], the effectiveness of MCARH171, a next-generation anti-BCMA CAR T cell, was investigated in patients with RRMM. This CAR T-cell incorporated a humanized anti-BCMA ScFv, CD3, 4-1BB, and a truncated epidermal growth factor receptor. This huEGFRt is functionally inert, meaning it lacks the N-terminal ligand-binding domains and receptor tyrosine kinase activity typical of the full-length EGFR. Despite these omissions, huEGFRt retains its native amino acid sequence, type I transmembrane localization, and a binding epitope that remains intact and recognizable by the anti-EGFR monoclonal antibody cetuximab (Erbitux). The utility of truncated protein is highlighted in several ways, as selection marker ex vivo and in vivo tracking. In addition, huEGFRt also serves as a target for cetuximab-mediated antibody-dependent cellular cytotoxicity. This allows for the potential elimination of the modified T cells in vivo, which can be an important safety feature in therapeutic applications [56].

Patients were administered four doses of CAR T-cells (72 × 10^6^, 137 × 10^6^, 475 × 10^6^, and 818 × 10^6^), resulting in a 64% overall response rate (ORR). However, a significant concern arose from the negative impact observed, as up to 40% of patients experienced grades 1–3 CRS [55]. Overall, the findings from this phase I trial provide promising insights into the potential of MCARH171 CAR T-cell therapy as a targeted approach for RRMM. Further investigations are warranted to refine the dosing regimen and develop strategies to mitigate CRS and other treatment-related toxicities, thereby enhancing the therapeutic efficacy and improving the long-term outcomes for patients with relapsed/refractory multiple myeloma [55, 57].

## BRD015 anti-BCMA CAR T cell

The effectiveness of BRD015, an anti-BCMA CAR T-cell therapy, was investigated in a phase I clinical trial (ChiCTR-OPC-16009113) involving 28 patients with RRMM [58, 59] BRD015 is composed of anti-BCMA ScFv, CD3ζ, and CD28 domains derived from mice. The therapy was administered at a dose ranging from 5.4 to 25.0 million CAR T-cells per kilogram. Among the patients who had high levels of BCMA expression on their multiple myeloma cells, an impressive overall response rate (ORR) of 87% was achieved [59]. This means that a vast majority of these patients experienced a significant reduction in tumor burden or even complete elimination of detectable cancer cells. In addition, a remarkable CR rate of 73% was observed in this subgroup, indicating a substantial and durable treatment response. Even more encouraging were the outcomes observed in patients with lower levels of BCMA expression on their tumor cells. This subgroup displayed an astounding 100% ORR, suggesting that BRD015’s anti-tumor activity extends beyond high BCMA expression levels. Although the complete response rate in this subgroup was slightly lower at 33%, the fact that all patients achieved a response is highly encouraging and highlights the potential broad applicability of BRD015 across a range of BCMA expression levels [59].

These findings from the phase I trial shed light on the promising therapeutic potential of BRD015 in RRMM [59]. The high response rates observed, both in patients with high and low BCMA expression, demonstrate the effectiveness of this novel CAR T-cell therapy in targeting multiple myeloma cells. Further studies and clinical trials are warranted to explore the long-term durability, safety profile, and potential combination therapies involving BRD015, with the aim of optimizing its efficacy and providing an innovative treatment option for patients with relapsed/refractory multiple myeloma.

## Descartes-08 anti-BCMA CAR T cell

Descartes-08 is an innovative CD8 + CAR T-cell product that has undergone genetic modification to express anti-BCMA CAR for a specific duration through mRNA transfection [60]. This controlled expression of anti-BCMA CAR for one week reduces the risk of uncontrolled proliferation.

One of the remarkable features of Descartes-08 is its high cytolytic activity against myeloma cells, irrespective of the presence of myeloma-protecting bone marrow stromal cells, exogenous proliferation-inducing ligands, or drug resistance, including resistance to IMiDs. In fact, in an aggressive disseminated human myeloma mouse model, Descartes-08 significantly suppresses myeloma growth that is specific to the BCMA CAR (p 0.0001) and greatly improves the survival of the host [60].

These promising preclinical results, combined with the ongoing clinical trial of Descartes-08 in relapsed/refractory myeloma patients (NCT03448978) [60], demonstrating preliminary durable responses and a favorable therapeutic index, have provided the foundation for the design of a recently initiated trial involving an optimized and humanized version of Descartes-08 known as Descartes-11. This trial aims to evaluate Descartes-11 in newly diagnosed myeloma patients with residual disease following induction therapy.

Furthermore, Escartes-08 exhibits notable efficacy against primary myeloma cells and MM cell lines, including those resistant to immunomodulatory drugs like lenalidomide and pomalidomide [60]. Encouraging results from a Phase I/II clinical trial in patients with relapsed/refractory multiple myeloma (NCT03448978) suggest the potential to achieve deep and long-lasting responses, such as stringent complete remission (sCR), without significant CAR T-cell-related toxicities like CRS or neurotoxicity. These findings support the recent completion of a Phase II study on Descartes-11, a humanized anti-BCMA mRNA CAR T-cell therapy, for frontline treatment of myeloma.

## CT103A anti-BCMA CAR T cell

CT103A BCMA-targeted CAR T cell, another next-generation CAR T cell, was tested in 18 patients with RRMM, including four who had previously been treated with a murine (mouse-origin) BCMA CAR therapy. (ChiCTR1800018137) [61, 62]. During the trial, patients received varying doses of CT103A CAR T-cells (1, 3, and 6 × 10^6^ CAR-positive T cells/kg) during the dose-escalation phase, and at 1 × 10^6^ CAR-positive T cells/kg in the expansion phase. The results obtained from this trial were exceptionally encouraging, as they demonstrated a remarkable overall response rate (ORR) of 100%. In addition, an impressive 72.2% of patients achieved a CR, indicating a substantial reduction or elimination of MM cells Among the four patients previously treated with murine BCMA CAR therapy, three achieved stringent complete response, and one achieved a very good partial response. The progression-free survival rate at one year was 58.3% for all cohorts, and 79.1% for patients without extramedullary myeloma. The most common adverse events were hematologic toxicities. About 70.6% of patients experienced grade 1 or 2 cytokine release syndromes. CAR transgenes were detectable in 77.8% of patients up to the cutoff date of the study, with a median persistence of 307.5 days. Only one patient developed antibodies against the therapy [61, 62].

## CD19-CAR T cell

CD19, a member of the immunoglobulin superfamily, plays a vital role as a multimolecular complex signaling component on mature B cells. In addition, it serves as a common target antigen expressed on various B cell hematological malignancies, including acute and chronic leukemias, as well as lymphomas [63]. The presence of CD19 on these malignancies makes it an attractive candidate for targeted therapy also in the treatment of MM though certain limitations are to be considered.

Multiple Myeloma is notably heterogeneous, characterized by significant variability in the cancer cells’ properties, such as antigen expression. While CD19 is typically absent in the majority of MM cells, it may be found in minor cell subsets with unique capabilities for propagating the disease. Addressing this, Garfall and colleagues embarked on a pioneering clinical trial (NCT02135406) to evaluate the efficacy of autologous T cells, engineered to express a chimeric antigen receptor (CAR) targeting CD19, referred to as CTL019 [64].

This innovative clinical trial was designed to synergize with standard MM therapy, integrating the use of CTL019 with autologous stem cell transplantation (ASCT) and high-dose melphalan. The objective was to explore the combined effect of these treatments in producing a significant clinical response in MM patients [64].

The study enrolled 12 patients with advanced-stage MM and aimed to evaluate the impact of this combination treatment approach on progression-free survival (PFS) rates. The results of the study were highly promising. The combination of ASCT, high-dose melphalan, and CTL019 therapy led to a significant increase in PFS rates for MM patients with advanced-stage disease [64]. This suggests that targeting CD19 with CTL019 in conjunction with the standard treatment regimen can potentially improve outcomes and extend survival in this patient population. The findings of this study highlight the potential of CD19 as a valuable therapeutic target to improve duration of response in the standard management of MM.

## CD19/BCMA CAR T cell

Fu et al. conducted a noteworthy clinical trial (NCT03196414) to explore the potential of third-generation CAR T-cell therapy in the treatment of RRMM. Their study aimed to evaluate the efficacy and safety of CAR T-cells engineered with an extracellular component comprising anti-BCMA and anti-CD19 single-chain variable fragments (ScFv). The CAR T-cells were further enhanced with a CD3 signaling domain and supplemented with CD28 and OX40 costimulatory molecules [65].

In this trial, a cohort of eight RRMM patients was enrolled, reflecting the pressing need for novel treatment options in this challenging disease setting. The patients received infusions of the genetically modified CAR T-cells, and the study team closely monitored their response to the therapy. By harnessing the specificity of anti-BCMA and anti-CD19 ScFv, along with the robust activation provided by the CD3 signaling domain and the additional costimulatory support from CD28 and OX40, the CAR T-cells aimed to effectively target and eliminate malignant plasma cells in the patients’ bodies. Moreover, another phase II clinical trial (ChiCTR-OIC-17011272) was carried out, where a group of twenty RRMM patients was treated with a combination therapy approach. This regimen involved the administration of both humanized anti-CD CAR T-cells (at a dosage of 1 × 10^6^ cells/kg) and murine anti-BCMA CAR T-cells (also at a dosage of 1 × 10^6^ cells/kg). By combining the two CAR T-cell therapies, the researchers sought to maximize the therapeutic response and potentially overcome any limitations associated with targeting a single antigen [66].

The results of this combined treatment approach were highly promising, as the study reported an impressive overall response rate (ORR) of 95%. This finding indicates that a significant majority of the RRMM patients experienced a positive response to the dual CAR T-cell therapy, underscoring the potential of such combination strategies in enhancing treatment outcomes.

## Kappa (κ) light chain-CAR T cell

Immunotherapy-based techniques have emerged as promising strategies for targeting specific subsets of light chains expressed on mature B cells. By leveraging the unique characteristics of light chains, these techniques enable the selective targeting of MM tumor cells in MM patients, while sparing healthy B cells. This targeted approach holds great potential for improving the efficacy and safety of MM treatments. In the context of immunoglobulins, it is worth noting that they are not typically expressed on the surface of plasma cells. However, evidence suggests that immunoglobulins may be expressed on MM stem cells, which are thought to play a crucial role in disease progression and relapse [67]. This finding opens up new possibilities for developing immunotherapeutic approaches that specifically target these elusive MM stem cells, potentially leading to more effective and durable responses. In their phase 1 clinical trial (NCT00881920), Ramos and colleagues treated 16 patients with relapsed or refractory κ+ non-Hodgkin lymphoma/chronic lymphocytic leukemia (NHL/CLL) or multiple myeloma (MM) [68]. Among the seven MM patients, four responded to the treatment. Notably, Patients 4 and 7 achieved stable disease (SD) for 17 and 24 months, respectively. Patient 4 reached minimal residual disease (MRD) post high-dose melphalan and autologous stem cell transplantation (ASCT), maintaining it for 17 months after κ.CART infusion. Patient 7 experienced reduced paraprotein levels and improved anemia for two years.

Patients 8 and 14 showed transient SD with significant paraprotein level changes. Patient 8, re-treated with κ.CART after 1.5 years and additional conventional therapy, achieved transient SD once more. However, the remaining three patients, including two who received chemotherapy just before κ.CART infusion, did not respond to the therapy. Overall, the study observed moderate anti-myeloma effects, such as decreased paraprotein levels, reduced free κ light chains, and improved anemia, lasting up to 24 months. The authors suggested that the modest responses in MM patients might be linked to low light chain expression on malignant plasma cells, and recommended further investigation into the distribution and persistence of κ.CARTs in the bone marrow of MM patients [68].

## NY-ESO-1-CAR T cell

In a preclinical investigation, engineered NY-ESO-1-CAR-T (or New York Esophageal Squamous Cell Carcinoma 1) cells were administered to a mouse model with NYESO-1/HLA-A2 MM, leading to notable tumor cell inhibition. This promising finding suggests the potential efficacy of NY-ESO-1-CAR-T cells in targeting NYESO-1-positive malignancies. Interestingly, the researchers also explored the combination of NYESO1-expressing synthetic T cells with membrane-bound IL-15, which demonstrated a synergistic effect. This combination not only enhanced anti-tumor cytotoxicity but also improved the persistence of memory CAR T-cells, providing a foundation for developing more effective immunotherapeutic strategies [69].

While the therapeutic potential of CAR T-cell therapies is immense, they can be accompanied by side effects. Reversible monocytopenia has been identified as a major concern associated with CD44v6-CAR T-cells. However, it is worth noting that this side effect may serve a beneficial purpose by preventing CRS By carefully balancing the modulation of immune response, it may be possible to leverage reversible monocytopenia to mitigate CRS while still maintaining the therapeutic benefits of CAR T-cells.

In addition to managing side effects, researchers have explored the integration of safety switches to enhance the safety profile of CAR T-cell therapies. Suicide genes, such as the thymidine kinase gene and the inducible caspase 9 gene, can act as safety switches to enable control over CAR T-cell activity. These genes can be engineered into CAR T-cells, allowing their activation or elimination when necessary. By incorporating such safety switches, the risk of adverse events and potential toxicity associated with CAR T-cell therapies can be reduced, offering an additional layer of safety and control [70].

These advancements in preclinical investigations not only shed light on the potential of NY-ESO-1-CART cells and combination therapies but also highlight the importance of addressing safety concerns in CAR T-cell therapies. With further research and development, these findings have the potential to pave the way for more effective and safer immunotherapeutic approaches in the treatment of cancer. While NY-ESO-1 has emerged as a promising target antigen in CAR-T therapy for Multiple Myeloma (MM), several critical limitations must be acknowledged. A key challenge is that NY-ESO-1 is not uniformly expressed across all MM cells. Its expression levels can significantly differ from one patient to another and may even vary among the cells within the same patient. Since NY-ESO-1 is only expressed in a subset of multiple myeloma cases, therapies targeting this antigen are not applicable to all patients with this disease. This limits the overall utility of NY-ESO-1-targeted therapies in the broader context of multiple myeloma treatment. A study evaluating LAGE-1 and NY-ESO-1 expression in MM patients found that NY-ESO-1 mRNA was expressed in only 26% of the MM samples analyzed by RT-PCR. In addition, both LAGE-1 and NY-ESO-1 protein expression were detected in just two cases by immunohistochemistry (IHC) [71].

It’s important to note that research is ongoing, and efforts are being made to address these limitations. This includes developing strategies to enhance CAR-T cell persistence, reduce side effects, and expand the range of targetable antigens in multiple myeloma.

## CD56-CAR T cell

Clinical trials, such as NCT03473496 and NCT03271632, have investigated the efficacy of CAR T-cell treatment targeting CD56, along with additional antigens expressed on MM cells. This approach aims to enhance the specificity and effectiveness of CAR T-cell therapy by targeting multiple antigens simultaneously. However, it is essential to consider the potential neurologic toxicity associated with CD56-CAR T-cells. Since CD56 is expressed not only on MM cells but also in both the central and peripheral nervous systems, there is a risk of adverse effects on the nervous system during CD56-CAR T-cell therapy [72].

## CD70-CAR T cell

CD70 (also known as CD27L) is a member of the tumor necrosis factor family and plays a crucial role in plasma cell development. While it is modestly expressed in healthy cells, CD70 has been found to be overexpressed in both solid tumors and hematological malignancies [73]. In the context of MM cases, various monoclonal antibodies (mAbs) such as BMS-936561 and SGN-75 have been developed to target myeloma cells. Notably, a preclinical study demonstrated that anti-CD70 CAR T-cells effectively and safely targeted CD70 + MM cells [74]. However, the use of anti-CD70 CAR T-cells in MM patients is currently limited due to the lower and variable expression of CD70 on MM cells [75].

## CD138-CAR T cell

CD138, also known as syndecan 1, is an adhesion molecule belonging to the syndecan family of heparan sulfate proteoglycans. It plays a crucial role in cell proliferation and the adhesion process by binding to collagen and fibronectin, which are extracellular matrix (ECM) molecules [76]. In MM, CD138 is often overexpressed, especially in relapsed or progressive cases, contributing to cancer progression [77]. Consequently, targeting CD138 holds promise as a potential therapeutic strategy for MM patients. One approach in treating MM patients involves the use of the anti-CD138 antibody-drug combination BT062 (indatuximab) as a treatment therapy. However, targeting CD138 with immune cells poses challenges due to CD138 shedding from MM cells, allowing them to evade the immune system. To address this issue, CD138-CAR T-cells could be utilized in conjunction with CAR T-cells targeting other antigens present on the surface of MM cells [78]. This combined approach could enhance the effectiveness of the treatment by targeting multiple antigens simultaneously.

Overall, CD138 presents an attractive target for therapy in MM, and further exploration of CD138-CAR T-cells and combination treatments holds promise in improving outcomes for MM patients.

## SLAMF7-CAR T cell

SLAMF7, also known as CD319 or CS1, is a transmembrane receptor belonging to the lymphocyte signaling activation molecule family [79]. It plays a crucial role in the phagocytosis of various hematological malignant cells by macrophages [75]. SLAMF7 is commonly found on immune cells such as CD4 and CD8 T cells, NK cells, activated B cells, plasma cells, dendritic cells, and monocytes [80]. Initially discovered as a receptor on NK cells [80], therapeutic antibodies targeting SLAMF7 have been developed, including the FDA-approved humanized monoclonal antibody, Elotuzumab (mAb). Elotuzumab, in combination with lenalidomide and dexamethasone, is used for the treatment of multiple myeloma patients [81]. In ongoing clinical trial research (NCT03710421), SLAMF7-CAR T-cells have been engineered by incorporating an anti-SLAMF7 single-chain variable fragment (ScFv), memory-enriched T cells, and a truncated EGFR (EGFRt) molecule to target multiple myeloma cells [82].

To address safety concerns associated with CAR T-cell therapy, a novel approach involving the use of cetuximab (an EGFR monoclonal antibody) has been explored. By employing cetuximab as an antibody-based safety switch, CAR T-cell suicide can be induced, thereby reducing the occurrence of severe immune-mediated adverse events following CAR T-cell administration, as previously mentioned [83].

Since SLAMF7 is not exclusively expressed on myeloma cells, therapies targeting this antigen may also affect other cells expressing SLAMF7. This can lead to unintended consequences or side effects, as healthy cells might be inadvertently targeted and damaged. The non-specificity of the antigen may result in a less effective treatment against myeloma cells. If the antigen is also present on normal cells, the therapy might not be as aggressive against cancer cells as it would be with a more specific target. There is a potential for the development of resistance. Cancer cells could adapt or mutate in a way that diminishes the efficacy of the treatment, especially if the therapy does not robustly discriminate between cancerous and non-cancerous cells. while targeting a non-specific antigen like SLAMF7 in myeloma has its limitations, such as the risk of off-target effects and potential resistance, it also offers opportunities for broader application and can be a valuable part of combination therapies. The impact of such approaches extends beyond immediate treatment efficacy, contributing to the ongoing evolution of cancer treatment paradigms [83].

## Anti-TACI CAR T cell

The transmembrane activator and CAML interactor (TACI), also known as TNFRSF13B, is a member of the tumor necrosis factor receptor (TNFR) superfamily [84]. It shares the activating ligands APRIL and BAFF with BCMA, promoting MM development and survival by binding to their respective receptors [84]. This led to the development of ligand-based CAR T-cells targeting APRIL, which simultaneously affect the TACI and BCMA signaling pathways [80, 81]. In an in vivo model, APRIL-directed CAR T-cells successfully eliminated BCMA + TACI and BCMATACI tumors, while monospecific anti-BCMA CAR T-cells were ineffective against BCMA cells, suggesting the potential use of APRIL-directed CAR T-cells in cases of BCMA loss [85, 86]. Trimeric APRIL-based CAR T-cells exhibited improved binding to BCMA and TACI receptors and demonstrated enhanced cytolytic activity compared to their monomeric counterparts.

In a recent study by Wong et al., non-viral gene delivery was employed to create BAFF CAR T-cells and a chimeric antigen receptor (CAR) based on BAFF ligands [87]. Through in vitro and in vivo experiments using various xenograft models, BAFF CAR T-cells specifically targeted the three BAFF receptors (BAFF-R, BCMA, and TACI) and effectively eliminated several B cell malignancies, including MM [87]. This ligand-based BAFF CAR T approach reduces the risk of antigen escape in the treatment of B cell malignancies by targeting three distinct receptors, including TACI [87].

Larson and colleagues developed a novel antibody against TACI that specifically recognizes TACI+ cells, excluding non-B cell peripheral blood mononuclear cells [88]. Using the antibody’s single chain variable fragment (scFv), they created second-generation CAR T-cells against TACI. In vitro, anti-BCMA CAR and anti-TACI CAR demonstrated comparable cytotoxicity against MM1S and RPMI-8226 multiple myeloma cell lines. Anti-TACI CARs also expanded in the peripheral blood of xenografted multiple myeloma animals and exhibited in vivo functionality [88]. The study indicated that the proximity of the anti-TACI scFv to the CD3 signaling domain influenced its effect on CAR functionality, with greater impact when closer to the domain [88].

TACI is present not only on myeloma cells but also on immunosuppressive regulatory T cells (Tregs) [89, 90]. Targeting TACI offers a two-pronged strategy, as it has the potential to indirectly manipulate the adverse milieu provided by Tregs and directly induce cytotoxicity through cytolysis [89].

CAR T-cell therapy has significantly improved the outlook for patients with recurrent/resistant hematologic malignancies, including acute lymphoblastic leukemia (ALL), chronic lymphocytic leukemia (CLL), non-Hodgkin lymphoma (NHL), and MM [12]. Table 1 provides a list of clinical trials evaluating CAR T-cell treatment for MM patients conducted at various centers.

**Table 1:** Major clinical trials using CAR-T cells in MM treatment.

ClinicalTrials. gov identifier and clinical trial phase	Study title	Ages eligible for study	Number of enrolled participants or patients	Center(s) or company	Reference
(NCT02658929) I	CRB-401 A Phase 1 Study of bb2121 in BCMA-Expressing Multiple Myeloma	18 Years and older	67 participants (33 patients)	Celgene	CT, [35]
(NCT03361748) II	Efficacy and Safety Study of bb2121 in Subjects with Relapsed and Refractory Multiple Myeloma (KarMMa)	18 Years and older	149 participants (100 patients)	Celgene	CT, [35, 37]
(NCT03601078) II	An Efficacy and Safety Study of bb2121 in Subjects with Relapsed and Refractory Multiple Myeloma and in Subjects with High-Risk Multiple Myeloma (KarMMa-2)	18 Years and older	235 participants	Celgene	CT, [38, 42]
(NCT03651128) III	Efficacy and Safety Study of bb2121 Versus Standard Regimens in Subjects with Relapsed and Refractory Multiple Myeloma (RRMM) (KarMMa-3)	18 Years and older	381 participants	Celgene	CT, [44]
(NCT04196491) I	A Study to Evaluate the Safety of bb2121 in Subjects with High Risk, Newly Diagnosed Multiple Myeloma (NDMM) (KarMMa-4)	18 Years and older	13 participants	Celgene	CT, [132]
(NCT04855136) I/II	Safety and Efficacy of bb2121 (Ide-cel) Combinations in Multiple Myeloma (KarMMa-7)	18 Years and older	415 participants	Celgene	CT, [45]
(NCT05032820) II	Assess anti-B Cell Maturation Antigen (BCMA) chimeric antigen receptor (CAR) T-cells (bb2121) to improve post autologous hematopoietic cell transplant (HCT) responses among patients with multiple myeloma (MM)	18 Years to 71 Years	40 participants	National Heart, Lung, and Blood Institute (NHLBI) Blood and Marrow Transplant Clinical Trials Network National Cancer Institute (NCI) National Marrow Donor Program Celgene a wholly owned subsidiary of BMS	NA
(NCT03274219) I	A Phase 1 Study of bb21217, an Anti-BCMA CAR T Cell Drug Product, in Relapsed and/or Refractory Multiple Myeloma	18 Years and older	72 participants	bluebird bio	CT, [47, 48]
(NCT03090659) I/II	LCAR-B38M Cells in Treating Relapsed/Refractory (R/R) Multiple Myeloma (LEGEND-2)	18 Years to 80 Years	100 participants (74 patients)	Nanjing Legend Biotech Co	CT, [50]
(NCT03288493) I/II	P-BCMA-101 Tscm CAR-T Cells in the Treatment of Patients with Multiple Myeloma (MM)	18 Years and older	105 participants	Poseida Therapeutics, Inc.	CT, [52]
(NCT03070327) I	BCMA Targeted CAR T Cells with or Without Lenalidomide for the Treatment of Multiple Myeloma	18 Years and older	20 participants	Memorial Sloan Kettering Cancer Center	CT, [133]
(NCT03448978) I/II	Autologous CD8 + T-cells Expressing an Anti-BCMA CAR in Patients With Myeloma	18 Years and older	38 participants	Cartesian Therapeutics	CT, [60]
(NCT02135406) I	Pilot Study Of Redirected Autologous T Cells Engineered To Contain Anti-CD19 Attached To TCRζ And 4-1BB Signaling Domains Coupled With Salvage Autologous Stem-Cell Transplantation (ASCT) In Multiple Myeloma Patients With Early Relapse/Progression After Initial ASCT	18 Years and older	13 participants (10 patients)	University of Pennsylvania	CT, [64]
(NCT03196414) I/II	Study of T Cells Targeting CD138/BCMA/CD19/More Antigens (CART-138/BCMA/19/More) for Chemotherapy Refractory and Relapsed Multiple Myeloma	18 Years to 75 Years	10 participants	The First Affiliated Hospital of Soochow University	CT, [117]
(NCT03473496) 0	CAR-T Cells Therapy in Relapsed/Refractory Multiple Myeloma (MM)	18 Years and older	2 participants	Zhujiang Hospital	NA
(NCT03271632) I/II	Multi-CAR T cell Therapy in the Treatment of Multiple Myeloma	18 Years to 80 Years	20 participants	Shenzhen Geno-Immune Medical Institute	NA
(NCT03710421) I	CS1-CAR T Therapy Following Chemotherapy in Treating Patients with Relapsed or Refractory CS1 Positive Multiple Myeloma	18 Years and older	30 participants	City of Hope Medical Center	NA
(NCT04657861) I	APRIL CAR-T Cell Therapy for Patients with BCMA/TACI Positive Relapsed and/or Refractory Multiple Myeloma	18 Years to 75 Years	36 participants	Zhejiang University	NA
(NCT05020444) I	TriPRIL CAR T Cells in Multiple Myeloma	18 Years and older	18 participants	Massachusetts General Hospital	NA
(NCT04133636) II	A Study of JNJ-68284528, a Chimeric Antigen Receptor T Cell (CAR-T) Therapy Directed against B-cell Maturation Antigen (BCMA) in Participants with Multiple Myeloma (CARTITUDE-2)	18 Years and older	157 participants	Janssen Research & Development, LLC	CT, [96]
(NCT04181827) III	A Study Comparing JNJ-68284528, a CAR-T Therapy Directed against B-cell Maturation Antigen (BCMA), Versus Pomalidomide, Bortezomib and Dexamethasone (PVd) or Daratumumab, Pomalidomide and Dexamethasone (DPd) in Participants with Relapsed and Lenalidomide-Refractory Multiple Myeloma (CARTITUDE-4)	18 Years and older	419 participants	Janssen Research & Development, LLC	CT, [96]
(NCT04923893) III	A Study of Bortezomib, Lenalidomide and Dexamethasone (VRd) Followed by Cilta-cel, a CAR-T Therapy Directed against BCMA Versus VRd Followed by Lenalidomide and Dexamethasone (Rd) Therapy in Participants with Newly Diagnosed Multiple Myeloma for Whom ASCT is Not Planned as Initial Therapy (CARTITUDE-5)	18 Years and older	650 participants	Janssen Research & Development, LLC	CT, [96]
(NCT05257083) III	A Study of Daratumumab, Bortezomib, Lenalidomide and Dexamethasone (DVRd) Followed by Ciltacabtagene Autoleucel versus Daratumumab, Bortezomib, Lenalidomide and Dexamethasone (DVRd) Followed by Autologous Stem Cell Transplant (ASCT) in Participants with Newly Diagnosed Multiple Myeloma (CARTITUDE-6)	18 Years and older	750 participants	European Myeloma Network	NA
(NCT04499339) I/II	A Phase I/IIa Clinical Trial to Assess Feasibility, Safety and Antitumor Activity of Autologous SLAMF7 CAR-T Cells in Multiple Myeloma	18 Years and older	38 participants	Wuerzburg University Hospital	NA
(NCT05016778) I	A Study of CAR-T Cells Targeting GPRC5D in the Treatment of r/r Multiple Myeloma	18 Years to 75 Years	15 participants	Zhejiang University	NA
(NCT04093596) I	Safety and Efficacy of ALLO-715 BCMA Allogenic CAR T Cells in Adults with Relapsed or Refractory Multiple Myeloma (UNIVERSAL) (UNIVERSAL)	18 Years and older	132 participants	Allogene Therapeutics	NA
(NCT04142619) I	Study Evaluating Safety and Efficacy of UCART Targeting CS1 in Patients with Relapsed/Refractory Multiple Myeloma (MELANI-01)	18 Years to 64 Years	18 participants	Cellectis S.A.	NA
(NCT03767751) I/II	A Feasibility and Safety Study of Dual Specificity CD38 and BCMA CAR-T Cell Immunotherapy for Relapsed or Refractory Multiple Myeloma	12 Years to 70 Years	80 participants	Chinese PLA General Hospital	NA
(NCT03287804) I/II	APRIL CAR T Cells (AUTO2) Targeting BCMA and TACI for the Treatment of Multiple Myeloma (APRIL)	18 Years and older	12 participants	Autolus Limited	NA
(NCT05066022) 0	A Study to Access the Safety and Efficacy of CT0590 in Patients with Relapsed and/or Refractory Multiple Myeloma	18 Years to 70 Years	24 participants	The First Affiliated Hospital of Soochow University	NA
(NCT05000450) I/II	Safety and Efficacy of ALLO-605 an Anti-BCMA Allogeneic CAR T cell Therapy in Patients with Relapsed/Refractory Multiple Myeloma	18 Years and older	136 participants	Allogene Therapeutics	NA
(NCT03674463) I	LCAR-B4822M-02 Cells in Treating Relapsed/Refractory (R/R) Multiple Myeloma	18 Years to 73 Years	10 participants	Second Affiliated Hospital of Xi’an Jiaotong University	NA
(NCT03751293) I	A Study of BCMA-directed CAR-T Cells Treatment in Subjects With r/r Multiple Myeloma	18 Years to 70 Years	10 participants	Hebei Yanda Ludaopei Hospital	CT, [134]
(NCT03318861) I	Study to Evaluate the Safety and Efficacy of KITE-585 in Participants with Relapsed/Refractory Multiple Myeloma	18 Years and older	17 participants	Gilead Sciences (Kite, A Gilead Company)	NA
(NCT03548207) Ib/II	A Study of JNJ-68284528, a Chimeric Antigen Receptor T Cell (CAR-T) Therapy Directed Against B-Cell Maturation Antigen (BCMA) in Participants With Relapsed or Refractory Multiple Myeloma (CARTITUDE-1)	18 Years and older	126 participants	Janssen Research & Development, LLC	CT, [96]
(NCT05201781) IV	A Long-term Study for Participants Previously Treated With Ciltacabtagene Autoleucel	18 Years and older	228 participants	Janssen Research & Development, LLC	CT, [97]
(NCT05172596) II	PHE885 CAR-T Therapy in Adult Participants with Relapsed and Refractory Multiple Myeloma	18 Years and older	136 participants	Novartis (Novartis Pharmaceuticals)	CT, [135]
(NCT04236011) I	BCMA and CD19 Targeted Fast Dual CAR-T for BCMA+ Refractory/Relapsed Multiple Myeloma	18 Years and older	15 participants	Gracell Biotechnology Shanghai Co., Ltd.	CT, [136]
(NCT04182581) I	A Study of BCMA/CD19 Dual-Target CAR-T Cell Immunotherapy for Relapsed or Refractory Multiple Myeloma	18 Years and older	18 participants	Gracell Biotechnology Shanghai Co., Ltd.	NA
(NCT03758417) II	A Study of LCAR-B38M CAR-T Cells, a Chimeric Antigen Receptor T-cell (CAR-T) Therapy Directed Against B-cell Maturation Antigen (BCMA) in Chinese Participants With Relapsed or Refractory Multiple Myeloma (CARTIFAN-1)	18 Years and older	130 participants	Janssen Research & Development, LLC	CT, [137]
(NCT03502577) I	BCMA-Specific CAR T-Cells Combined With a Gamma Secretase Inhibitor (JSMD194) to Treat Relapsed or Persistent Multiple Myeloma	21 Years and older	18 participants	Juno Therapeutics, Inc.	CT, [138]
(NCT05722418) I	CRISPR-Edited Allogeneic Anti-BCMA CAR-T Cell Therapy in Patients with Relapsed/Refractory Multiple Myeloma (CaMMouflage)	18 Years and older	50 participants	Caribou Biosciences, Inc.	CT
(NCT04318327) I	Updated phase I study results of PHE885, a T-Charge manufactured BCMA-directed CAR-T cell therapy, for patients (pts) with r/r multiple myeloma (RRMM)	18 Years and older	96 participants	Novartis Pharmaceuticals	CT, [102, 103]
(NCT05535244) I/II	A Study Evaluating the Efficacy and Safety of Cevostamab in Prior B cell Maturation Antigen (BCMA)-Exposed Participants with Relapsed/Refractory Multiple Myeloma (CAMMA 2)	18 Years and older	140 participants	Hoffmann-La Roche	CT, [104]
(NCT05066646) I/II	A Phase 1/2 Study of a Fully Human BCMA-targeting CAR (CT103A) in Patients with Relapsed/Refractory Multiple Myeloma (FUMANBA-1) (FUMANBA-1)	18 Years and older	132 participants	Nanjing IASO Biotherapeutics Co.,Ltd	CT, [105]

## CAR T-cell FDA-approved

Currently, six CAR T-cell treatments have been authorized by the FDA: Tisagenlecleucel (Kymriah), Axicabtagene Ciloleucel (Yescarta), Brexucabtagene Autoleucel (Tecartus), Lisocabtagene Maraleucel (Breyanzi), Idecabtagene Vicleucel (Abecma), and iltacabtagene autoleucel (Carvykti) [91].

The FDA recently approved ciltacabtagene autoleucel (Carvykti), also known as cilta-cel, for adults who have either treatment-resistant or relapsed multiple myeloma. This groundbreaking approval was granted on February 28, 2022 [92–95]. Ciltacabtagene autoleucel incorporates two single-domain antibodies that specifically target B-cell maturation antigen. It is indicated for patients who have undergone four or more lines of therapy, including treatment with three major types of multiple myeloma medications: an anti-CD38 monoclonal antibody, a proteasome inhibitor, and an immunomodulating agent. This approval provides new hope for patients who have exhausted these treatment options. Various clinical trials utilizing ciltacabtagene autoleucel are currently underway to investigate its efficacy in treating patients with relapsed or refractory multiple myeloma (NCT03548207, NCT04133636, NCT04181827, NCT04923893, NCT03548207) [96].

The ongoing clinical trial CARTITUDE-1 (NCT03548207) investigates the use of cilta-cel in patients with multiple myeloma who have received multiple prior therapies. This study served as the basis for FDA clearance [93, 97, 98]. In the trial, each of the 97 participants received a single infusion of cilta-cel. Nearly all of them (98 percent) responded to the treatment, indicating a partial reduction in cancer burden. Impressively, 78 percent of individuals showed no evidence of cancer in their bone marrow or blood, demonstrating a complete response. In patients with RRMM who have undergone extensive prior treatments, a single infusion of cilta-cel resulted in deeper and durable responses with a manageable safety profile at a median follow-up of 2 years (MFU). The two CAR T-cell products licensed by the FDA (ide-cel and cilta-cel) target the BCMA protein on the surface of myeloma cells and have shown efficacy in treating multiple myeloma. Furthermore, several meta-analyses conducted by Costa et al. using available indirect treatment comparison (ITC) data demonstrated that cilta-cel provides a significant advantage over physician’s choice of treatment (PCT) for patients with triple-class exposed RRMM [99]. These findings highlight the efficacy of cilta-cel as a therapeutic option for patients with triple-class exposed RRMM [99]. In general, the long-term outcomes of patients with triple-refractory multiple myeloma treated with BCMA CAR T-cell therapies ide-cel and cilta-cel in the KarMMa and CARTITUDE-1 trials have demonstrated improved outcomes for patients with poor prognoses [100].

## CAR T-cell therapy limitations for MM

Despite achieving high response rates in several BCMA-targeted CAR T-cell therapies, the durability of responses remains a therapeutic challenge in the treatment of MM. A significant proportion of patients experience relapse [89], indicating the complex nature of CAR T-cell therapy failure in MM. This failure can be attributed to a combination of patient-specific factors, characteristics of the malignancy itself, and immune-related variables, which are analogous to those observed in CD19 + B lymphoid malignancies.

One of the key underlying mechanisms of relapse after cellular immunotherapy is the emergence of tumors with low or negative antigen expression, thereby evading CAR T-cell elimination. This phenomenon, known as antigen escape, has been observed in patients who relapse following CAR T-cell treatment. Notably, the expression of B-cell maturation antigen (BCMA), the target antigen for CAR T-cell therapy in MM, has been found to be downregulated or lost in these relapsed patients [101–103]. Unlike CD19+ lymphoid malignancies, no DNA alterations have been reported as contributing factors in MM relapses, suggesting alternative mechanisms at play.

Another potential mechanism that could result in lower target antigen density is CAR T-cell-mediated trogocytosis. This process involves the removal of malignancy-associated surface proteins from tumor cells through direct contact with lymphocytes. It has been suggested that CAR T-cells can strip off BCMA or other targeted antigens from the surface of malignant plasma cells via trogocytosis [104]. This phenomenon may contribute to the reduced levels of target antigen available for CAR T-cell recognition and subsequent elimination.

In addition to antigen-related factors, the lack of CAR T-cell persistence has been identified as another aspect contributing to recurrence in MM patients. The transient presence of CAR T-cells in the body limits their ability to provide sustained therapeutic effects. Efforts are being made to enhance the persistence of CAR T-cells through various strategies, including genetic modifications and the use of co-stimulatory molecules, in order to improve treatment outcomes [105].

Furthermore, the immunosuppressive effects of the tumor microenvironment (TME) and malignant plasma cells within MM patients may play a significant role in their resistance to immune-based therapy. The TME, characterized by its unique composition and interactions, can create a hostile environment that hampers the effectiveness of CAR T-cells. Factors such as the presence of inhibitory immune checkpoints, immunosuppressive cytokines, and regulatory immune cells can impede CAR T-cell function and limit their ability to eliminate malignant plasma cells effectively [88, 106].

Understanding and addressing these multifaceted mechanisms of CAR T-cell therapy failure in MM is crucial for improving treatment outcomes and achieving long-term remissions. Ongoing research efforts are focused on developing strategies to enhance target antigen expression, improve CAR T-cell persistence, and overcome immunosuppressive barriers within the tumor microenvironment. These advancements hold promise for the future of CAR T-cell therapy in the management of multiple myeloma.

## Clinical CAR T-cell manufacturing in MM

The remarkable success observed in early-phase clinical studies with CD19-targeted CAR T-cells for treating hematologic malignancies has generated significant interest in CAR T cell-based therapies [22]. Currently, there is a growing focus on targeting different types of tumors by incorporating additional tumor-associated antigens such as PSMA, mesothelin, GD2, HER2, and epidermal growth factor receptors. This area of research is highly active and numerous clinical trials are underway [107].

While the designs and tumor-specific single-chain variable fragments (scFvs) may vary, the manufacturing technique for CAR T-cells remains consistent. The process involves collecting and processing T-cell sources, followed by CAR T-cell preparation which includes T-cell selection and/or activation, genetic modification using a CAR cDNA, large-scale expansion, and end-of-process formulation. To ensure the quality of the final product, in-process and quality control release testing is closely integrated into the production process. In clinical trials, CAR T-cells derived from the CD3+ population are commonly used [107]. However, recent studies from various laboratories have demonstrated that specific subsets of T cells, such as naive, central memory, and memory stem cells, may possess functional advantages. Consequently, techniques for the clinical-scale selection, transduction, and expansion of these T-cell subsets have also been developed [107]. Although it is tempting to initiate CAR T-cell manufacturing with specific T-cell populations, there is still a need to establish which T-cell subsets offer the best therapeutic efficacy, least toxicity, and are compatible with a robust and reproducible manufacturing process [107].

## Challenges of CAR T-cell therapy for MM

### Tumor antigen heterogeneity

Multiple Myeloma (MM) presents a significant challenge in oncology due to its complex tumor antigen heterogeneity, a critical aspect of the disease’s pathology. This malignancy is primarily characterized by monoclonal plasma cell proliferation, stemming from cells with identical variable-diversity-joining (VDJ) recombination at immunoglobulin loci [108]. Such malignancy demonstrates notable variability, both genomically and transcriptomically, varying significantly across patients. This heterogeneity, influenced by diverse clonal evolution patterns and a complex tumor microenvironment, creates substantial challenges in understanding and addressing the impact of polyclonality on tumor progression and patient outcomes [108].

Further complicating MM’s pathology is its extensive inter-patient genomic heterogeneity, resulting from various initiating events [109]. Insights from multi-region sequencing studies highlight spatial differences within MM, with progression events such as TP53 mutations often confined to specific focal lesions. This degree of heterogeneity is a significant factor in clinical outcomes, influencing everything from prognostic categorization to therapeutic approach and response assessment [109].

Intratumor Heterogeneity (ITH) in MM also plays a crucial role in these clinical outcomes [108]. High ITH is associated with tumor immune escape and resistance to treatment. While the connection between neoantigens and ITH is recognized, the specific details and functional implications of this relationship require further elucidation [110].

MM is further characterized by both inter- and intratumoral heterogeneity [111]. It undergoes a clonal evolutionary process influenced by clonal competition, the tumor microenvironment, host immunity, and therapy. Cytogenetically, MM is divided into two primary groups: one with recurrent translocations at the immunoglobulin heavy chain locus, and the other characterized by hyperdiploidy involving odd-numbered chromosomes. The disease typically initiates with a preneoplastic phase, known as monoclonal gammopathy of undetermined significance, and can progress to symptomatic MM over variable periods [111].

This tumor heterogeneity, evolving over time and space, has significant implications for clinical management, including challenges in disease classification, risk stratification, and during maintenance therapy, where clonal evolution can complicate monitoring and contribute to drug resistance. The identification and understanding of dominant neoplastic clones are essential for personalized therapy, especially in cases of disease progression or transformation.

These insights into MM’s tumor antigen heterogeneity highlight the necessity for a comprehensive research and treatment approach. This approach should integrate genetic, molecular, and clinical data to better understand and tackle the complexities of this multifaceted disease.

### Immunosuppressive tumor microenvironment

The presence of a tumor microenvironment characterized by an abundance of cells and inhibitory chemicals can significantly impede the effectiveness of CAR T-cell therapy. To overcome this challenge, extensive research efforts have been dedicated to enhancing the performance of CAR T-cells under adverse conditions by modifying their metabolic profiles [112].

In malignancies, high levels of adenosine and reactive oxygen species (ROS) often impact T cell responses. Cancer-associated fibroblasts (CAFs), which are prominent components of the tumor microenvironment, express a significant amount of fibroblast activation protein (FAP) and play a critical role in establishing an immunosuppressive milieu. They also release extracellular matrix (ECM) proteins that hinder T cell infiltration in solid tumors [113].

While the mechanism through which anti-CTLA-4 antibodies enhance endogenous T cell responses to cancer is still uncertain, their potential to augment CAR T-cell responses is an intriguing area of exploration. In addition, by reducing CTLA-4+ Treg cells, anti-CTLA-4 antibodies may trigger an immune response in a cell-extrinsic manner, thereby potentially aiding CAR T-cells in their function [114].

Circumventing the immunosuppressive microenvironment in cancer therapy, Alabanza et al. in 2022, put forth the innovative concept of ‘armored CAR T cells.’ This design involves engineering CAR T cells to combat TGF-β-induced suppression, a common hurdle in effective cancer treatment. By integrating a BCMA-targeting CAR with a specialized DN-TGF-βIIR ‘armor,’ these armored CAR T cells gain an enhanced ability to withstand TGF-β‘s suppressive effects, even under prolonged exposure. This breakthrough in CAR T cell design is particularly significant for improving treatment efficacy and durability in multiple myeloma (MM) patients [115].

## Clinical challenge of CAR T-cell therapy for MM

### CAR T-cell treatment toxicity profiles

Cytokines and other immunological proteins released from the infused cells and/or host cells, such as macrophages, contribute to the toxicities associated with CAR T-cell therapy [116] The elevation of inflammatory mediators in the bloodstream, including IL-6, TNF, and IFN, among others, leads to acute onset CRS [116]. While fever is the most common symptom of CRS, it can also manifest as tachycardia, hypotension, hypoxia, and other symptoms [116]. Neurological toxicities are another significant side effect of CAR T-cells, which can present as headache, aphasia, delirium, and, less frequently, seizures and obtundation due to cerebral edema [116]. Hematologic toxicity following CAR T-cell infusion is complex and multifactorial, involving lymphodepleting chemotherapy, inflammation, HLH, infections, disease recurrence, and subsequent neoplasms, including myelodysplastic syndrome [116].

### ICANS

Neurotoxicity events, such as immunological effector cell-associated neurotoxicity syndrome (ICANS), can occur following CAR T-cell infusion. These events can vary in nature and manifest with a wide range of clinical presentations [116–118]. Signs of ICANS may include aphasia, altered consciousness, cognitive impairment, motor weakness, seizures, and cerebral edema. It is believed that ide-cel and cilta-cel, which carry 4-1BB domains, pose a reduced risk and severity of ICANS compared to CD28-carrying CAR T-cell products. The slower T-cell expansion caused by these domains is thought to mitigate the severity of ICANS [116–118]. One of the an important, albeit rare, fatal case has been reported in the study by Van Oekelen et al. on a case involving a patient with multiple myeloma (MM) who was treated with BCMA-targeting CAR-T cell therapy as part of the CARTITUDE-1 trial [119]. Approximately three months after receiving ciltacabtagene autoleucel, a BCMA-targeted CAR-T cell infusion, the patient developed a progressive movement disorder with parkinsonism features. This condition was associated with the persistence of CAR-T cells in the blood and cerebrospinal fluid and lymphocytic infiltration in the basal ganglia. The study also found BCMA expression on neurons and astrocytes in the patient’s basal ganglia. This observation, along with public transcriptomic datasets showing BCMA RNA expression in the caudate of normal human brains, suggests that the neurological symptoms might be an on-target effect of anti-BCMA therapy. The paper highlights the importance of close neurological monitoring for patients undergoing BCMA-targeted T cell therapies, especially considering reports of similar parkinsonism symptoms in other patients treated with this therapy [119].

### Dual CAR-T cells to improve MM specific

**Fig. 4:**
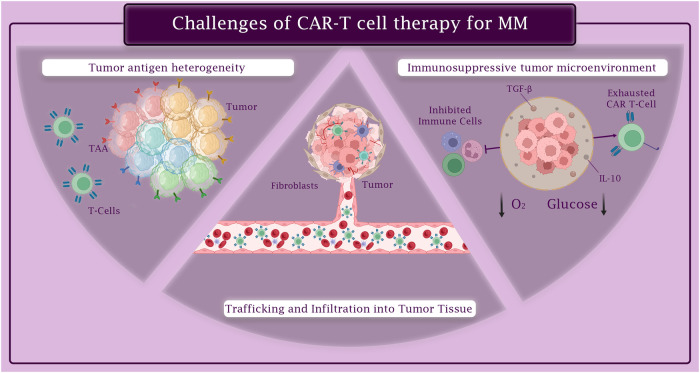
Challenges of CAR-T cell therapy for MM. Challenges of CAR-T cell therapy for MM include: Tumor antigen heterogeneity, Immunosuppressive tumor microenvironment, and Trafficking and infiltration into tumor tissue.

Dual-targeting with different CAR T-cells offers the advantage of individually controlling CAR expression in each CAR T-cell product (Fig. 4). Moreover, this technique enables sequential delivery of CAR T-cells, which may reduce the risk of severe CRS [120]. In recent clinical trials involving newly diagnosed and relapsed refractory patients, BCMA/CD19 dual CAR T-cell targeting was achieved by using two pools of single CAR Transduced T-cells (NCT03706547, NCT03767725). To minimize the potential for exacerbated cytokine release syndrome, patients received CAR T-cells in a sequential manner. Both groups of patients experienced tolerable CRS without any neurological damage. Evaluation of clinical responses demonstrated that sequential combination of BCMA and CD19 CAR T-cells could enhance therapeutic outcomes [120–122]. However, since these studies lacked a control group (i.e., a single-arm design), it is crucial to examine the results of a recently launched study (NCT03549442) that investigates the comparative success and safety of dual-targeting BCMA/CD19 as opposed to BCMA targeting alone. Clinical trials are currently underway to explore dual CAR T-cell targeting with combinations of BCMA/CD19 and BCMA/SLAMF7, utilizing bicistronic vectors (NCT04156269, NCT04162353) [120]. Several other clinical trials employing non-specific dual targeting techniques are investigating BCMA/CD38, BCMA/NY-ESO1, and CD38/CD19 combinations (NCT03125577, NCT03767751, NCT03473496, NCT03271632, and NCT03638206). In the clinic, BCMA/GPRC5D dual CAR T-cells are being tested through co-infusion, co-transduction, or creation using a bicistronic vector [122]. In addition, inhibitory CARs (iCARs) are used in another type of dual CAR design to enhance specificity. The iCAR method, first described by Fedorov et al. in 2013, involves combining a CAR T-cell with a standard second-generation CAR, which has a scFv binding domain coupled with an inhibitory cytoplasmic domain such as CTLA-4 and PD-1 [123] (Fig. 4B).

### Split-dual CAR T-cells to improve MM-specificity

**Fig. 5:**
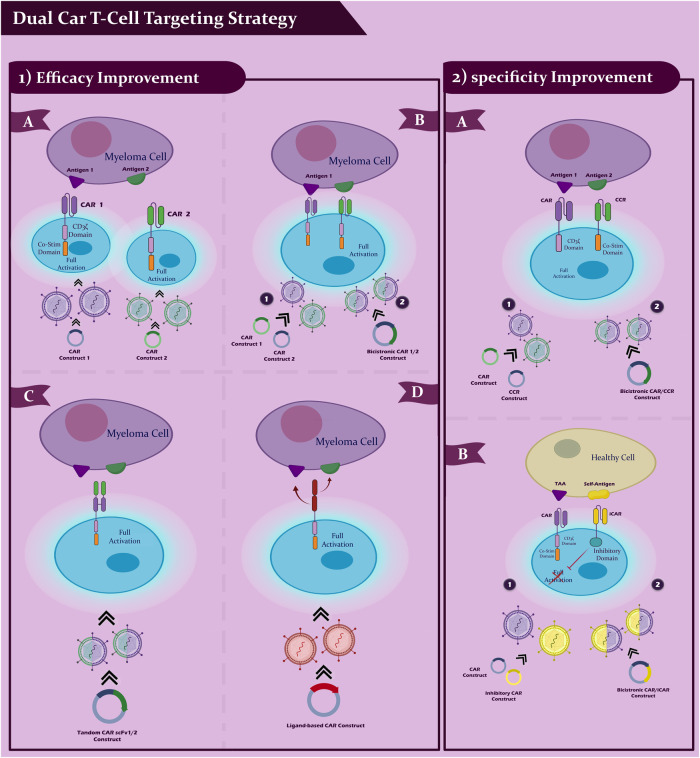
Strategies to overcome challenges to MM. Dual CAR T-cell targeting is one of the essential strategies for targeting MM using CAR T-cell, which includes improvement in efficacy and specificity. (1) Efficacy improvement. **A** Dual CAR: depicts a myeloma cell expressing two antigens (Antigen 1 and Antigen 2); two separate CAR T cells, infused together or sequentially, each with a distinct CAR (CAR 1 and CAR 2), target each antigen; the co-stimulatory domain and CD3ζ domain in both CARs lead to complete activation of the T cells upon binding to their respective antigens, destroying the cancer cell. **B** Bicistronic CAR (1/2 construct): a single T cell with a bicistronic CAR construct can express two different CARs; full T cell activation occurs upon dual binding, suggesting improved efficacy through bispecific targeting. **C** Tandem CAR (scFv1/2 construct): a single T cell with a tandem CAR construct containing two binding sites (scFv1 and scFv2) for the two antigens; binding of both sites results in full T cell activation, suggesting another approach for enhancing efficacy through tandem CAR design. **D** ligand-based CAR construct: a T cell with a ligand-based CAR construct is shown to recognize and bind to the ligand; upon binding, full activation of the T cell occurs, indicating that ligand-based targeting can also improve the efficacy of CAR T-cell therapy. (2) Specificity improvement. **A** Bicistronic CAR/CCR construct: a T cell with a bicistronic CAR construct that includes a CAR and a co-stimulatory receptor (CCR) is shown; the T cell requires binding to both antigens for full activation, suggesting that dual antigen recognition can improve specificity and reduce off-target effects. **B** Inhibitory CAR (iCAR): shows a healthy cell with a TAA (tumour-associated antigen) and a self-antigen not present in the tumour cells; a T cell with a dual construct: a CAR for the TAA and an inhibitory CAR (iCAR) for the self-antigen; if the T cell encounters a healthy cell that expresses the self-antigen, the iCAR sends an inhibitory signal to prevent T cell activation, enhancing the specificity of the therapy by avoiding damage to healthy cells.

Split-dual CAR technology utilizes two separately expressed CARs targeting carefully selected antigens, which individually may not be tumor-specific but demonstrate tumor-specific expression when combined (Fig. 5). The primary activation and co-stimulation signals for T-cells are divided between these two CARs. This ensures that dual CAR Transduced T-cells are fully activated only when both CARs engage their targets on tumor cells simultaneously, while avoiding recognition of a single antigen on normal tissues. By splitting the initial and co-stimulatory T-cell activation signals, split-dual CARs enhance tumor specificity (Fig. 5) [124].

While the main objective of split-dual CAR T-cells is to improve specificity, they can also enhance the affinity of CAR T-cells for the tumor target. BCMA, for instance, exhibits limited expression in healthy cells except for plasma cells, making split signaling less necessary to enhance BCMA CAR T-cell specificity. However, incorporating MM antigens that are not exclusive to plasma cells and myeloma cells, particularly those strongly expressed on MM cells but with lower expression on healthy cells (e.g., CD138, CD38, and SLAMF7), can offer advantages and open new possibilities [125]. Combining a non-specific MM target with an iCAR targeting a self-antigen could enhance the specificity and safety of MM CAR therapy. Split-signaling CAR T-cells and dual CAR/iCAR T-cells can be generated through the co-transduction of two independent vectors or the use of a bicistronic vector, each with its own advantages and disadvantages [126].

To enhance the antimyeloma efficacy of CAR T-cell therapy and prevent myeloma cells from evading treatment through antigen loss and downregulation, dual-antigen targeting of BCMA and other myeloma antigens such as SLAMF7, CD19, and GPRC5D has been proposed [127, 128]. A novel approach to dual-antigen targeting involves not only targeting the tumor cells but also eradicating the cancer-associated fibroblasts (CAFs) that create the myeloma niche in the bone marrow, as demonstrated by Sakemura et al. [129]. In preclinical models, both in vitro and in vivo, T-cells directed against BCMA combined with either SLAMF7 or fibroblast-associated protein (FAP) exhibited significantly improved antimyeloma efficacy. These enhanced CAR T-cells displayed greater cytolytic activity, cytokine production, and proliferation against multiple myeloma cells in the presence of CAFs, surpassing the performance of standard CAR T-cells targeting BCMA as a single antigen [130].

### Strategies to overcome clinical challenges to MM

Anti-IL-6 receptor antibodies, such as Tocilizumab, along with corticosteroids, have been widely utilized in the treatment of CRS. However, when it comes to managing neurological toxicities, corticosteroids are commonly favored over anti-IL-6 receptor antibodies [131]. In an effort to minimize potential side effects associated with this type of therapy, CAR T-cell products are now being developed with genes that incorporate a biological suicide switch. This innovative feature allows for the activation of the suicide switch and subsequent induction of CAR T-cell apoptosis following the administration of a specific medication infusion.

The incorporation of a biological suicide switch in CAR T-cell therapy holds promise for addressing safety concerns. By providing a controlled means of eliminating CAR T-cells when necessary, this technology offers an additional layer of safety and control. The suicide switch can be triggered in response to specific circumstances, such as the occurrence of severe adverse events or the completion of the desired therapeutic effect. This ensures that CAR T-cells can be selectively removed from the patient’s system, reducing the risk of potential long-term complications or adverse reactions.

The activation of the suicide switch prompts a programmed cell death mechanism, leading to the apoptosis of CAR T-cells. This process involves the activation of genes encoding proteins that induce cell death pathways, such as apoptosis-inducing receptors or pro-apoptotic enzymes. Once activated, these proteins initiate a cascade of events that ultimately result in the targeted elimination of CAR T-cells. By employing this precise mechanism, the biological suicide switch offers a reliable and controllable way to modulate the activity of CAR T-cells and mitigate potential toxicities associated with their prolonged presence.

The introduction of a suicide switch in CAR T-cell therapy not only enhances safety but also provides reassurance to patients and healthcare providers. The ability to deactivate CAR T-cells if necessary alleviates concerns about uncontrolled cellular proliferation, immune overactivation, or potential long-term effects. It empowers clinicians to intervene promptly and manage adverse events effectively, fostering a more personalized and tailored approach to CAR T-cell therapy.

In conclusion, the integration of a biological suicide switch into CAR T-cell products represents an exciting advancement in the field of immunotherapy. This innovation holds promise for improving the safety profile of CAR T-cell therapies, particularly by reducing the potential side effects associated with prolonged CAR T-cell activity. By enabling the selective elimination of CAR T-cells when needed, the suicide switch technology provides an additional layer of control and enhances the overall effectiveness and safety of CAR T-cell therapy.

## Conclusion and future perspectives

Despite recent breakthroughs, the treatment options for patients with multiple myeloma remain limited and ineffective. One promising approach is the use of CAR T-cells, which are engineered T cells equipped with lymphocyte-like signaling molecules. These cells have shown potential in targeting specific genes that are preferentially expressed in malignant cells, making them a potential therapeutic avenue for boosting the immune system’s response against cancerous cells.

If we can fully understand the potency of CAR T-cell therapy in controlling multiple myeloma over the long term, it could have a disruptive effect in the field of cancer treatment. Clinical trials involving CAR T-cells in hematological malignancies are already underway, exploring thousands of combinations of therapies in this area. However, one challenge lies in finding more effective targets for CAR T-cell therapy and identifying suitable combination therapies.

To decrease the risk of relapse in multiple myeloma after CAR T-cell therapy, researchers are focusing on novel antigens that can be targeted either alone or in combination with B-cell maturation antigen (BCMA). In addition, pharmacological drugs can be used to enhance the density of target antigens on multiple myeloma cells, potentially improving clinical outcomes. Modifying the design of CAR T-cells by altering the antigen-binding, co-stimulatory, hinge, and transmembrane domains can also enhance their effectiveness. Furthermore, incorporating a suicide switch system into CAR T-cell therapy can enhance overall safety.

Another approach to improving the effectiveness of CAR T-cell therapy is the addition of maintenance therapy following CAR T-cell infusion or replacing standard lymphodepleting chemotherapy regimens with more potent drugs against multiple myeloma. By combining these strategies, researchers hope to achieve more durable and profound responses in patients.

**Fig. 6:**
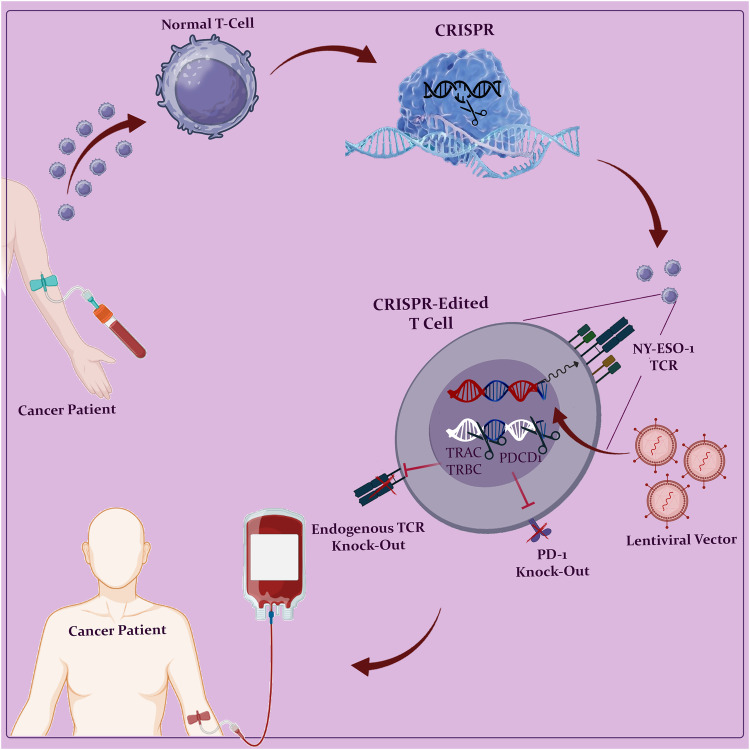
Gene editing of T cells using of CRISPR-Cas9 strategy in cancer patients. A cancer patient’s blood was used to isolate T cells. The CRISPR-Cas9 strategy was used for gene editing in normal T cells. After injecting the patient with the gene-edited T cells again, patients were observed to determine the efficacy and safety of the treatment.

In order to advance the field of CAR T-cell therapy, it is crucial to conduct basic mechanistic research, alongside bioengineering and various clinical trials. Such research provides insights into the fundamental biology of T cells and helps in the development of “fitter” CAR T-cells that exhibit increased proliferation and durability. The emergence of CRISPR-Cas9 technology has opened up new possibilities, allowing for genome-wide screening to identify novel genes that, when modified, can enhance the resilience and capabilities of CAR T-cells (Fig. 6).

Furthermore, understanding the heterogeneity of multiple myeloma and the distinct genomes and transcriptomics of cancer cells could provide valuable information on how different surface targets can be utilized for immune targeting. Single-cell analysis of plasma cell leukemia patients has already revealed the presence of multiple subsets of CAR T-cells with varying expression, proliferation, and cytotoxicity characteristics, indicating stage-specific changes in their development. Exploring the influence of specific genomic and transcriptomic features, such as T-cell classes and RNA editing signatures, on the expression of surface targets could shed light on new avenues for immune targeting in multiple myeloma.
